# Dual hypothermic oxygenated machine perfusion of the liver reduces post-transplant biliary complications: a retrospective cohort study

**DOI:** 10.1097/JS9.0000000000002115

**Published:** 2024-10-18

**Authors:** David Pereyra, Jule Dingfelder, Moriz Riha, Sertac Kacar, Laurin Rauter, Nikolaus Becker, Tina Saffarian Zadeh, Chiara Tortopis, Patrick Starlinger, Robin Ristl, Gerd Silberhumer, Andreas Salat, Thomas Soliman, Gabriela Berlakovich, Georg Gyoeri

**Affiliations:** a Department of General Surgery, Division of Transplantation, Medical University of Vienna, General Hospital of Vienna; b Department of General Surgery, Division of Visceral Surgery, Medical University of Vienna, General Hospital of Vienna, Vienna, Austria; c Department of Surgery, Division of Hepatobiliary and Pancreas Surgery, Mayo Clinic, Rochester, Minnesota, USA; d Center for Medical Data Science, Institute of Medical Statistics, Medical University of Vienna, Vienna, Austria

**Keywords:** biliary complications, hypothermic machine perfusion, liver transplantation

## Abstract

**Background:**

Corroborating evidence for the use of hypothermic oxygenated machine perfusion (HOPE) prior to orthotopic liver transplantation (OLT) suggests a beneficial effect in regard to biliary complications. Here, the authors aim to evaluate whether perfusion via portal vein alone (sHOPE) or via additional perfusion of the hepatic artery (dHOPE) have diverging impact on outcomes after OLT when compared to the use of static cold storage (SCS).

**Methods:**

Consecutive patients undergoing OLT at the Medical University of Vienna (2018–2023) were retrospectively analyzed. Donor organs were procured using SCS, or subjected to end-ischemic sHOPE or dHOPE. The severity of biliary complications was classified according to the degree of therapeutic intervention (endoscopic retrograde cholangiopancreatography or surgical revision).

**Results:**

Two hundred forty-seven patients were included (69 SCS, 76 sHOPE, and 102 dHOPE). Hospitalization was shorter for patients after HOPE (median in days: SCS=25 vs HOPE=20, *P*=0.019). Biliary complications were less frequent in patients after HOPE (SCS=37.7% vs HOPE=22.5%, *P*=0.015). A significantly lower incidence of surgical revisions for biliary complications was observed in the HOPE cohort (24.6% vs 11.8%, *P*=0.012). When evaluating outcome according to HOPE-modality, a significant reduction in biliary complications (*P*=0.006) and surgical revisions (*P*=0.002) was only observed in dHOPE patients in comparison to SCS. Further, only dHOPE was significantly associated with a reduced need for surgical revision for biliary complications upon univariable and multivariable logistic regression (odds ratio=0.336, *P*=0.011).

**Conclusion:**

HOPE leads to a reduction of biliary complications and associated surgical revisions. This effect seems to be primarily associated with use of dHOPE, while both methods appear as feasible options for preconditioning of donor grafts prior to OLT.

## Introduction

Highlights

- Use of HOPE is associated with reduced incidence of biliary complications and surgical revisions.
- Only dHOPE is associated with the reduction of surgical reinterventions for biliary complications upon multivariable analysis.

Machine perfusion of procured organs prior to orthotopic liver transplantation (OLT) has led to a paradigm shift in the field^1^. Both, normothermic and hypothermic perfusion devices are currently used in clinical routine with the goal to evaluate and precondition marginal grafts^2^. Despite the increasing use of extended criteria donors for OLT, the concommitent high risk for postoperative complications cannot be neglected^3^. In particular, biliary complications occur frequently and impact patients short-term and long-term outcomes.

A multicenter analysis of over 2000 low-risk benchmark cases undergoing OLT at European and American high-volume centers revealed an incidence of biliary complications of 20%^4^. When evaluating outcome after OLT with marginal grafts, the proportion of patients developing biliary complications increases drastically.

Corroborating evidence suggests a beneficial role of hypothermic oxygenated machine perfusion (HOPE) prior to OLT on patient outcome^2,5^. Pathophysiologically oxygenation under hypothermic conditions was shown to reduce ischemia-reperfusion injury through replenishment of intracellular adenosine triphosphate and controlled succinate degradation, thereby limiting mitochondrial damage^6–8^. Randomized controlled trials showed a reduction of biliary complications via the use of HOPE^9–12^. Interestingly, oxygenation and perfusion via the portal vein alone (i.e. single HOPE=sHOPE) was proposed to suffice for the induction of protective pathways during HOPE^13^. However, simultaneous perfusion via the hepatic artery and portal vein (i.e. dual HOPE=dHOPE) is generally feasible using commercially available devices. Of note, the use of either sHOPE or dHOPE seems to rely on center preference, while there is currently no data on direct comparison of both modalities^14^. The here presented retrospective observational study aimed to evaluate the relevance of HOPE-modalities for outcome after OLT when compared to preservation via SCS, while specifically focusing on the incidence and type of biliary complications in a real-world setting of consecutive patients undergoing OLT.

## Methods

### Patients

All subsequent patients undergoing OLT at the Medical University of Vienna between May 2018 and August 2023 and receiving organs after SCS or HOPE were included in this retrospective cohort study. This included high-urgency indications and retransplantations. As transplantation of split liver grafts are not routinely performed at our center, all patients in this cohort underwent full-graft OLT. No patient in the present cohort underwent multiorgan transplantation. The present study was approved by the institutional ethics committee of the Medical University of Vienna (EK#1610/2023) and was conducted in accordance with the Declaration of Helsinki (as revised in 2013). All patients gave written informed consent. The study was registered on ClinicalTrials.gov (identifier: NCT06418165). No funding was received for this study and the authors do not have conflicts of interest associated with this investigation.

### Organ procurement and hypothermic machine perfusion

All organs were procured using standardized operating procedures and transported to the transplant unit using static cold storage (SCS). During multiorgan procurement and SCS, histidine-tryptophan-ketoglutarate (HTK, Custodiol) solution was used. After backtable preparation of donor grafts, HOPE was initialized, or SCS continued. Either dHOPE or sHOPE were applied whenever logistically possible and regardless of donor characteristics, as described below. Of note, reasons to not perform sHOPE or dHOPE were mostly the lack of a dedicated perfusionist or technical issues with the perfusion machine prior to perfusion (i.e. maintenance periods). The decision on whether to use sHOPE or dHOPE was made at surgeons discresion and dependent on the availability of an aortic patch or the suitability of arterial vessels for canulation (e.g. multiple arteries and need of complex backtable reconstruction). HOPE was performed with the LiverAssist Perfusion System (XVIVO Perfusion) and Belzer University of Wisconsin machine perfusion solution (UW-MPS) was used during this step. Perfusion was carried out at 8–15°C. The device perfuses the grafts with a continuous flow through the portal vein and with a pulsatile flow (60 bpm) through the hepatic artery. The flow is pressure dependent thus rising and falling with changing pressure, which can be adjusted in 1 mmHg steps. This results in optimal flow through the liver and enables gentle reperfusion of the graft tissue. The Liver Assist has two hollow fiber membrane oxygenators to oxygenate the perfusion fluid. HOPE was ultimately applied for the time of hepatectomy and duration varied accordingly. Subsequently, the liver was flushed with Belzer University of Wisconsin (UW) solution prior to organ implantation. Ultimately, an albumin flush was performed prior to the completion of caval anastomosis in order to reach near-physiological electrolyte concentrations prior to reperfusion.

Of note, cold ischemia time was defined as time of SCS, which encompasses the entire time of organ preservation in the SCS cohort, while it was the time until start of perfusion in the sHOPE and dHOPE cohort. In addition, total time of preservation (i.e. cold ischemia time + perfusion) is given for organs preserved via either HOPE-modality.

### Postoperative follow-up and biliary complications

During follow-up, the development of complications, time in the ICU, and total time of hospitalization were documented. Of note, there is no intermediate care unit for patients after OLT at our department. Hence, all patients were transferred to an ICU ward immediately after the operation. After discharge, patients were followed-up at the transplant outpatient clinic within standardized intervals. Routine magnetic resonance cholangiopancreatography (MRCP) was performed four months after OLT. In addition, MRCP was performed when indicated, that is, in case of symptoms related to ischemic-type biliary lesions or cholangitis. All types of biliary complications and time of occurrence were documented in the prospectively maintained institutional database and categorized according to the type of complication in bile leak, anastomotic stricture, and nonanastomotic stricture. In addition, the severity of biliary complication was further classified according to treatment: need for either intervention via endoscopic retrograde cholangio-pancreatography (ERCP) or any type of surgical revision (drainage and lavage, bile duct revision, and hepaticojejunostomy).

### Definition of outcome

Early allograft dysfunction (EAD) was defined and evaluated according to the criteria described by Olthoff *et al*.^15^. Briefly, one of the following criteria had to be present to fulfill Olthoff’s criteria for EAD: bilirubin ≥10 mg/dl on POD7, international normalized ratio (INR) ≥1.6 on POD7, alanine or aspartate aminotransferases (ALAT/ASAT) >2000 IU/L within the first seven PODs. Patients fulfilling any of these criteria were referred to as ‘EAD’, whereas patients not included by these criteria were defined as ‘no EAD’.

Morbidity was prospectively documented as the incidence of every occurrence deviating from the normal postoperative course. Comprehensive complication index (CCI) was used as a metric measurement of morbidity and calculated for each patient as previously described^16^. Briefly, all complications were graded according to the criteria given by Dindo *et al*.^17^, weighted and summarized in a score from 0 to 100 points, whereby the overall burden of complications is estimated. After discharge, patients were followed-up at the institutional outpatient clinic. Survival was prospectively documented. Further, death-censored graft survival was evaluated and defined as time until retransplantation or death due to graft dysfunction versus death with functioning liver graft.

### Statistical analyses

Statistical analyses were performed using SPSS version 27.0 (SPSS, Inc.) and R version 4.2 and were based on nonparametric testing. The distribution of metric variables between the two groups was compared using Wilcoxon signed-rank tests. The *χ* ^2^ test was used for comparison of categorical variables. Univariate and multivariable logistic binomial regression were applied for the evaluation of associations between baseline and donor characteristics and type of organ procurement with patient outcome in regards to biliary complications with the need for surgical intervention. Survival rates and time to first biliary complication were estimated by the Kaplan–Meier method and compared between groups using log-rank tests. In addition, a competing risk analysis was conducted with biliary complication, re-transplantation, and death as competing events. Cumulative incidence curves were calculated for each group and compared using Gray’s test. *P*-values <0.05 considered as statistically significant. Box plots are visualized without significant outliers in order to improve resolution of interquartile distributions. This work is reported in accordance with the strengthening the reporting of cohort, cross-sectional, and case–control studies in surgery (STROCSS) (Supplemental Digital Content 1, http://links.lww.com/JS9/D514) criteria^18^.

## Results

### Patient demographics

A total of 247 procured liver grafts, which were subsequently transplanted, were included in this study. Of these, 69 (27.9%) were preserved via SCS, while the remaining 178 organs were subjected to HOPE. In detail, sHOPE was performed in 76 (30.8%) of liver grafts and dHOPE in 102 (41.3%) organs. Recipient and donor demographics are visualized in Table 1. The majority of included individuals underwent first OLT and median postoperative follow-up was 19.4 months. Of note, the incidence of cardiovascular comorbidities was significantly elevated in recipients receiving SCS organs, and subjects in the sHOPE cohort displayed increased BMI and reduced use of DCD organs. Of note, most liver grafts included in this cohort were procured from donors after brain death. Differences in these baseline demographics are visualized in Supplementary Figure 1 (Supplemental Digital Content 2, http://links.lww.com/JS9/D515). Further, cold ischemic time was significantly shorter for liver grafts subjected to either sHOPE or dHOPE, as time on the pump was not defined as cold ischemic time. However, total preservation time (i.e. cold ischemic time + perfusion time in HOPE cohorts or entire preservation time in SCS organs) was comparable between all subgroups. The median duration of machine perfusion was 150 min and did not differ between sHOPE and dHOPE (Table 1).

**Table 1:** Recipient and donor demographics.

Parameter	Entire cohort (*N*=247)	SCS (*N*=69)	sHOPE (*N*=76)	dHOPE (*N*=102)	*P* ^a^	*P* ^b^	*P* ^c^
Recipient							
Male (*n*, %)	193 (78.1)	49 (71.0)	60 (78.9)	84 (82.4)	0.568	0.080	0.568
Age, years (median, IQR)	57.0 (49.0–63.0)	56.0 (48.5–62.5)	56.5 (47.3–62.0)	58.5 (49.0–64.0)	0.849	0.472	0.349
MELD, points (median, IQR)	16 (11–21)	16 (12–22)	15 (11–20)	16 (11–20)	0.416	0.637	0.690
MELD-Na, points (median, IQR)	16 (12–21)	18 (13–25)	16 (11–21)	17 (12–20)	0.080	0.067	0.933
Platelet count, G/l (median, IQR)	94 (65–139)	92 (68–141)	94 (73–129)	97 (60–145)	0.958	0.593	0.773
Creatinine, mg/dl (median, IQR)	0.92 (0.77–1.20)	0.94 (0.73–1.22)	0.92 (0.81–1.11)	0.93 (0.80–1.27)	0.866	0.556	0.385
Bilirubin, mg/dl (median, IQR)	2.19 (1.11–4.27)	1.78 (1.02–4.06)	2.33 (1.22–4.49)	2.21 (1.05–4.27)	0.288	0.838	0.406
GGT, U/l (median, IQR)	97 (56–188)	122 (53–219)	89 (54–163)	97 (60–185)	0.302	0.802	0.420
INR (median, IQR)	1.5 (1.3–1.8)	1.5 (1.3–1.8)	1.5 (1.3–1.8)	1.5 (1.3–1.7)	0.942	0.675	0.578
BMI, kg/m^2^ (median, IQR)	25.6 (23.1–29.9)	24.8 (21.6–29.4)	26.1 (23.0–29.6)	26.3 (23.8–30.6)	0.174	**0.027**	0.348
First OLT (*n*, %)	232 (93.9)	63 (91.3)	72 (94.7)	97 (95.1)	0.415	0.321	0.913
HU-OLT (*n*, %)	15 (6.1)	5 (7.6)	3 (4.0)	7 (6.9)	0.360	0.861	0.415
Follow-up, months (median, IQR)	19.4 (7.7–35.2)	20.0 (3.9–35.8)	23.3 (11.0–34.8)	15.1 (7.7–36.9)	0.660	0.934	0.519
Cardiovascular disease (*n*, %)	91 (36.8)	35 (51.5)	23 (30.3)	33 (32.7)	**0.010**	**0.015**	0.733
Arterial hypertension (*n*, %)	82 (33.2)	24 (34.8)	23 (30.3)	35 (34.3)	0.561	0.950	0.568
Diabetes type II (*n*, %)	45 (18.2)	13 (18.8)	13 (17.1)	19 (18.6)	0.786	0.972	0.794
Smoking (*n*, %)	31 (12.6)	6 (8.7)	13 (17.1)	12 (11.9)	0.134	0.507	0.323
Ascites (*n*, %)	84 (34.0)	25 (36.2)	26 (34.2)	33 (32.4)	0.799	0.599	0.795
Encephalopathy (*n*, %)	39 (15.8)	13 (18.8)	10 (13.2)	16 (15.7)	0.350	0.590	0.637
Hepatorenal syndrome (*n*, %)	20 (8.1)	5 (7.2)	6 (7.9)	9 (8.8)	0.883	0.712	0.825
Chronic kidney disease (*n*, %)	34 (13.8)	7 (10.1)	9 (11.8)	18 (17.6)	0.745	0.173	0.286
Pulmonary diseases (*n*, %)	44 (17.8)	11 (15.9)	18 (23.7)	15 (14.7)	0.244	0.825	0.127
Donor							
Male sex (*n*, %)	135 (54.7)	33 (47.8)	44 (57.9)	58 (56.9)	0.471	0.397	0.471
DCD (*n*, %)	10 (4.0)	5 (7.7)	4 (5.5)	1 (1.0)	0.599	**0.025**	0.082
Age, years (median, IQR)	53.0 (43.0–63.0)	51.0 (42.8–61.3)	53.0 (43.3–64.0)	55.0 (41.5–63.0)	0.820	0.421	0.534
BMI, kg/m^2^ (median, IQR)	25.0 (23.0–28.0)	24.0 (22.0–27.3)	25.0 (23.0–28.0)	25.0 (23.0–28.0)	0.286	0.235	0.915
Smoking (*n*, %)	67 (27.1)	23 (33.3)	20 (26.3)	24 (23.5)	0.243	0.467	0.635
Diabetes type II (*n*, %)	16 (6.5)	4 (5.8)	5 (6.6)	7 (6.8)	0.838	0.607	0.7532
Alcohol abuse (*n*, %)	23 (9.3)	5 (7.3)	8 (10.5)	10 (9.8)	0.458	0.344	0.845
DRI (median, IQR)	1.7 (1.4–2.0)	1.7 (1.5–2.0)	1.7 (1.4–2.0)	1.6 (1.3–1.9)	0.529	0.196	0.507
ET-DRI (median, IQR)	1.6 (1.4–1.9)	1.7 (1.5–2.0)	1.6 (1.4–1.9)	1.6 (1.4–1.9)	0.370	0.451	0.847
GGT, U/l (median, IQR)	36 (21–88)	36 (17–89)	32 (20–73)	43 (25–100)	0.582	0.145	**0.027**
Steatosis, % (median, IQR)	4 (0–10)	5 (0–15)	2 (0–10)	2 (0–10)	0.264	0.202	0.877
Cold ischemia, min (median, IQR)	319 (255–410)	453 (392–569)	305 (235–345)	283 (221–334)	**<0.001**	**<0.001**	0.234
Perfusion time, min (median, IQR)	150 (105–223)	–	150 (87–229)	150 (120–210)	–	–	0.414
Total preservation time, min (median, IQR)	443 (379–539)	453 (392–569)	434 (356–559)	443 (382–498)	0.301	0.293	0.825

a SCS vs sHOPE.

b SCS vs dHOPE.

c sHOPE vs dHOPE.

Bold values indicate statistical significance with *p*<0.05.

DRI, donor risk index; ET, Eurotransplant; GGT, gama-glutamyltransferase; HOPE, hypothermic oxygenated machine perfusion; HU, high urgency; INR, international normalized ratio; IQR, interquartile range; MELD, model of end-stage liver disease; OLT, orthotopic liver transplantation; SCS, static cold storage.

*P* values.

### Impact of HOPE-treatment on hospitalization, EAD, and in-hospital complications

**Figure 1:**
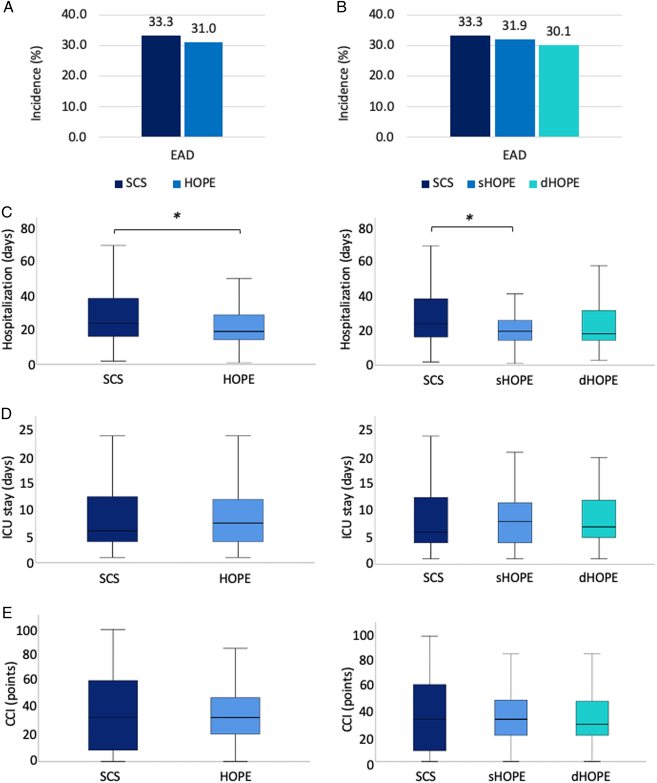
Hospitalization is reduced in patients after HOPE. (A) Incidence of early allograft dysfunction (EAD) is visualized for patients after static cold storage (SCS) and for hypothermic oxygenated machine perfusion (HOPE) subgroups (SCS: 18 of 54 [33.3%], HOPE: 44 of 142 [31.0%]; *P*=0.752), and (B) between SCS and HOPE-modalities (sHOPE: 22 of 69 [31.9%], dHOPE: 22 of 73 [30.1%]; SCS vs sHOPE: *P*=0.865, SCS vs dHOPE: *P*=0.701, sHOPE vs dHOPE: *P*=0.822). (C) Hospitalization is shorter for patients after HOPE (median in days: SCS=25 vs HOPE=20, *P*=0.019), while there is no difference between sHOPE and dHOPE (median in days: sHOPE=21 vs dHOPE=19; SCS vs sHOPE: *P*=0.018, SCS vs dHOPE: *P*=0.065, sHOPE vs dHOPE: *P*=0.543). There are no differences between cohorts in regards to (D) ICU stay (median in days: SCS=6 vs HOPE=8, *P*=0.476; median in days: sHOPE=8 vs dHOPE=7, SCS vs sHOPE: *P*=0.860, SCS vs dHOPE: *P*=0.322, sHOPE vs dHOPE: *P*=0.516), and (E) comprehensive complication index (CCI; median: SCS=33.7 vs HOPE=33.5, *P*=0.596; median: SCS=33.7 vs sHOPE=33.7 vs dHOPE=29.6, SCS vs sHOPE: *P*=0.986, SCS vs dHOPE: *P*=0.408, sHOPE vs dHOPE: *P*=0.313). *P<0.05.

The incidence of EAD was 31.6% in the entire cohort. No difference in the development of EAD was observed between SCS and HOPE (*P*=0.752; Fig. 1A). Further, there was no difference between modalities of HOPE in regards to frequency of EAD (SCS vs sHOPE: *P*=0.865, SCS vs dHOPE: *P*=0.701, sHOPE vs dHOPE: *P*=0.822; Fig. 1B). Hospitalization was significantly shorter for patients undergoing OLT after HOPE (*P*=0.019; Fig. 1C), while no difference was observed for stay at ICU (*P*=0.476; Fig. 1D) and CCI (*P*=0.596; Fig. 1E). No difference in hospitalization, ICU stay and CCI was observed between sHOPE and dHOPE (Fig. 1C–E). Of note, there was no difference in postoperative laboratory parameters associated with liver damage and function, as visualized in Supplementary Table 1 (Supplemental Digital Content 2, http://links.lww.com/JS9/D515).

### HOPE is associated with a decreased incidence of biliary complications after OLT

**Figure 2:**
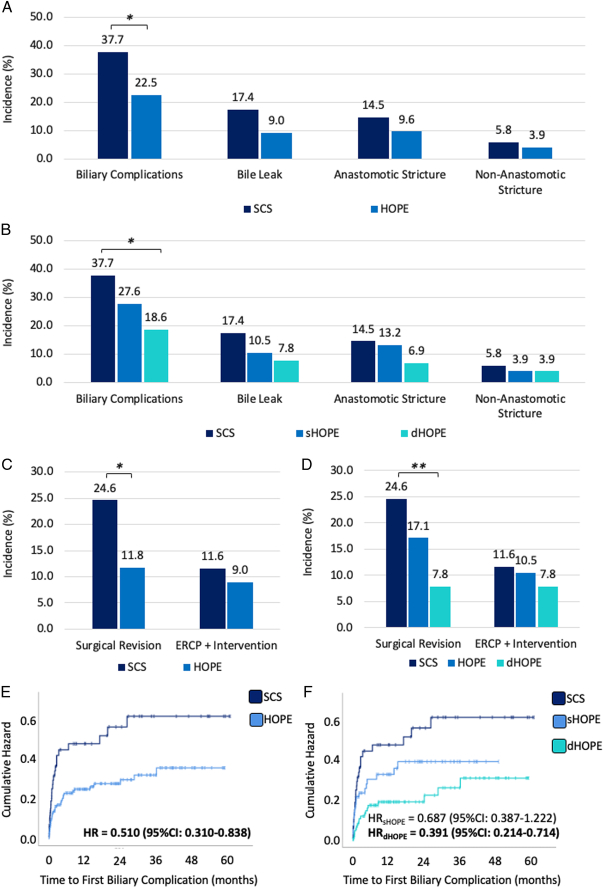
Biliary complications and associated surgical revisions are reduced in patients after HOPE. (A) Incidence of all biliary complications (SCS: 26 of 69 [37.7%], HOPE: 40 of 178 [22.5%]; *P*=0.015), as well as bile leaks (SCS: 12 of 69 [17.4%], HOPE: 16 of 178 [9.0%], *P*=0.062), anastomotic stenosis (SCS: 10 of 69 [14.5%], HOPE: 17 of 178 [9.6%], *P*=0.264), and nonanastomotic stenosis (SCS: 4 of 69 [5.8%], HOPE: 7 of 178 [3.9%], *P*=0.524) are shown for patients after static cold storage (SCS) and hypothermic oxygenated machine perfusion (HOPE). (B) Similarly, incidence of these complications is shown for SCS in comparison to HOPE-modalities (all biliary complications: 21 of 76 [27.6%] in sHOPE, 19 of 102 [18.6%] in dHOPE, sHOPE vs SCS: *P*=0.197, dHOPE vs SCS: *P*=0.006, sHOPE vs dHOPE: *P*=0.155; bile leak: 8 of 76 [10.5%] in sHOPE, 8 of 102 [7.8%] in dHOPE, sHOPE vs SCS: *P*=0.231, dHOPE vs SCS: *P*=0.057, sHOPE vs dHOPE: *P*=0.536; anastomotic strictures: 10 of 76 [13.2%] in sHOPE, 7 of 102 [6.9%] in dHOPE, sHOPE vs SCS: *P*=0.816, dHOPE vs SCS: *P*=0.102, sHOPE vs dHOPE: *P*=0.158; nonanastomotic strictures: 3 of 76 [3.9%] in sHOPE, 4 of 102 [3.9%] in dHOPE, sHOPE vs SCS: *P*=0.604, dHOPE vs SCS: *P*=0.569, sHOPE vs dHOPE: *P*=0.993). (C) Incidence of surgical revision (SCS: 17 of 69 [24.6%], HOPE: 21 of 178 [11.8%], *P*=0.012) and interventional endoscopic retrograde cholangio-pancreatography (ERCP, SCS: 8 of 69 [11.6%], HOPE: 16 of 178 [9.0%], *P*=0.535) is shown for patients after SCS and HOPE. (D) Similarly, incidences of these events are shown for SCS in comparison to HOPE-modalities (surgical revision: 13 of 76 [17.1%] in sHOPE, 8 of 102 [7.8%] in dHOPE, sHOPE vs SCS: *P*=0.263, dHOPE vs SCS: *P*=0.002, sHOPE vs dHOPE: *P*=0.058; interventional ERCP: 8 of 76 [10.5%] in sHOPE, 8 of 102 [7.8%] in dHOPE, SCS vs sHOPE: *P*=0.838; SCS vs dHOPE: *P*=0.409; sHOPE vs dHOPE: *P*=0.536;). Hazard plots for occurrence of first biliary complication after liver transplantation are shown for (E) static cold storage (SCS) and hypothermic oxygenated machine perfusion (HOPE) (mean time to complication in months: SCS=36.1, 95% CI: 28.4–43.8; HOPE=45.2, 95% CI: 41.0–49.5, log-rank: *P*=0.007; Cox-regression: hazard ratio [HR]=0.510, 95% CI: 0.310–0.838, *P*=0.008) and (F) for HOPE-modalities (mean time to complication in months: sHOPE=35.2, 95% CI: 29.8–40.7, dHOPE=48.0, 95% CI: 42.7–53.2, log-rank: SCS vs sHOPE *P*=0.194, SCS vs dHOPE *P*=0.001, sHOPE vs dHOPE *P*=0.070; Cox-regression: sHOPE - HR=0.687, 95% CI: 0.387–1.222, *P*=0.202, dHOPE - HR=0.391, 95% CI: 0.214–0.714, *P*=0.002). *P<0.05, **P<0.005.

Biliary complications, defined as the development of either bile leak, anastomotic strictures, or nonanastomotic strictures, occurred after 66 of 247 transplantations (26.7%). In detail, 28 bile leaks (11.3%), 27 anastomotic strictures (10.9%), and 11 nonanastomotic strictures (4.5%) were observed. A lower incidence of biliary complications was observed in the HOPE cohort (37.7% vs 22.5%, *P*=0.015; Fig. 2A). Intriguingly, this effect was pronounced in dHOPE treated organs (27.6% in sHOPE, 18.6% in dHOPE; sHOPE vs SCS: *P*=0.197; dHOPE vs SCS: *P*=0.006; Fig. 2B), while there was no statistically significant difference between sHOPE and dHOPE grafts (*P*=0.155; Fig. 2B). When consequently evaluating types of biliary complications, use of dHOPE displayed a tendency for reduction of biliary leaks (dHOPE vs SCS: *P*=0.057; Fig. 2B). No difference in the incidence of anastomotic strictures or nonanastomotic strictures were observed. A comparison of the incidence of biliary complications without biliary leaks can be found in Supplementary Figure 2 (Supplemental Digital Content 2, http://links.lww.com/JS9/D515).

### dHOPE specifically reduces the need for surgical revisions of biliary complications

In this cohort, 24 complications (42.4% of all biliary complications) were managed via interventional ERCP, while a total of 38 surgical revisions (57.6% of all biliary complications) were conducted. In detail, 22 patients received hepatico-jejunostomy, 14 patients underwent reanastomisis of the bile duct, one patient received a T-drain, and lavage and drainage was performed in one case. Use of HOPE was associated with a significant reduction in surgical revisions for biliary complications (*P*=0.012; Fig. 2C), while no difference in the use of ERCP with intervention was observed (*P*=0.535; Fig. 2C). When evaluating the effect of HOPE-modalities in regards to surgical revisions for biliary complication, a prominent reduction of 16.8% was observed for dHOPE-treatment when compared to SCS (*P*=0.002; Fig. 2D). This effect was not observed for the use of sHOPE (*P*=0.263; Fig. 2D). In fact, there was still a trend towards reduced surgical revisions in dHOPE treated organs when using sHOPE as a comparator (*P*=0.058). No differences between groups were observed for biliary complications managed via ERCP with intervention (Fig. 2D). Comparison of incidence of biliary complications according to severity without biliary leaks can be found in Supplementary Figure 2 (Supplemental Digital Content 2, http://links.lww.com/JS9/D515).

In order to evaluate the impact of HOPE-modality on the development of biliary complications with the requirement of surgical revision, univariable logistic regression identified the use of dHOPE and transplantation of DCD grafts as significant parameters, while there was no association of sHOPE treatment with this outcome parameter (Table 2). When evaluating both parameters in a multivariable logistic regression, dHOPE-treatment prior to OLT remained as the only independent variable in the model (Table 2). Of note, a second multivariable logistic regression model including parameters being significantly altered in baseline demographics while not reaching statistical significance upon univariate logistic regression can be found in Supplementary Table 2 (Supplemental Digital Content 2, http://links.lww.com/JS9/D515). Here, stepwise forward exclusion was used, and use of dHOPE was confirmed as the only independent variable associated with the development of biliary complications with the requirement of surgical revision. Similarly, dHOPE remained independently associated with the development of any type of biliary complication upon univariable and multivariable logistic regression (Supplementary Table 3, Supplemental Digital Content 2, http://links.lww.com/JS9/D515).

**Table 2:** Multivariable analysis for biliary complication with surgical intervention.

	Univariate analysis			Multivariable analysis		
Parameter	OR	95% CI	*P*	OR	95% CI	*P*
Use of sHOPE	1.205	0.579–2.507	0.618			
Use of dHOPE	**0.326**	**0.143**–**0.745**	**0.008**	**0.336**	**0.146**–**0.775**	**0.011**
Perfusion time (min)*	0.999	0.994–1.005	0.810			
Recipient characteristics						
Male	1.131	0.499–2.561	0.768			
Age (years)	1.012	0.980–1.045	0.459			
MELD (points)	1.029	0.235–1.079	0.235			
MELD-Na (points)	1.027	0.980–1.075	0.266			
BMI (kg/m^2^)	1.000	0.931–1.074	0.995			
First OLT	0.000	NA	0.998			
HU-OLT	2.075	0.624–6.896	0.234			
Cardiovascular diseases	1.184	0.580–2.419	0.643			
Arterial hypertension	0.916	0.436–1.925	0.818			
Diabetes type II	1.240	0.526–2.921	0.623			
Smoking	0.789	0.259–2.398	0.676			
Ascites	0.652	0.300–1.415	0.279			
Encephalopathy	0.585	0.195–1.753	0.338			
Hepatorenal syndrome	0.590	0.131–2.652	0.491			
Chronic kidney disease	0.307	0.070–1.340	0.116			
Pulmonary disease	1.281	0.543–3.024	0.571			
Donor characteristics						
Male sex	1.086	0.538–2.194	0.818			
DCD	**3.804**	**1.020**–**14.191**	**0.047**	2.871	0.752–10.957	0.123
Age (years)	0.986	0.966–1.008	0.207			
BMI (kg/m^2^)	0.924	0.843–1.013	0.094			
Smoking	0.739	0.308–1.774	0.499			
Diabetes type II	0.673	0.145–3.131	0.614			
Alcohol abuse	1.311	0.445–3.864	0.623			
DRI	1.000	0.998–1.002	0.951			
ET-DRI	0.726	0.195–2.696	0.632			
GGT (U/l)	1.000	0.997–1.004	0.912			
Steatosis (%)	0.993	0.961–1.025	0.646			
Cold Ischemia (min)	1.002	0.999–1.005	0.129			

*Univariate analysis of perfusion time is restricted to the HOPE cohort (i.e. sHOPE and dHOPE).

Bold indicate statistical significance with *p*<0.05.

DCD, donation after cardiac death; DRI, donor risk index; ET, Eurotransplant; GGT, gama-glutamyltransferase; HOPE, hypothermic oxygenated machine perfusion; HU, high urgency; MELD, model of end-stage liver disease; Na, sodium; OLT, orthotopic liver transplantation; OR, odds ratio.

### Time to the first biliary complication is prolonged in dHOPE-treated organs

In addition to the frequency and type of biliary complication, the time until the first complication was evaluated. Here, HOPE-treated organs displayed a later occurrence (on average 36.1 vs. 45.2 months, *P*=0.007). This difference was particularly strong when evaluating time to first biliary complication according to the modality of HOPE, where dHOPE-treated organs seemed to experience a mean delay of roughly 1 year when compared to SCS (SCS vs sHOPE: *P*=0.194, SCS vs dHOPE: *P*=0.001, sHOPE vs dHOPE: *P*=0.070). This difference is visualized in the hazard plots for SCS versus HOPE (Fig. 2E) or versus HOPE-modalities (Fig. 2F). Cox-regression revealed a reduction of risk for biliary complications in patients treated after any type of HOPE [hazard ratio (HR)=0.510, 95% CI: 0.310–0.838, *P*=0.008; Fig. 2E]. However, when evaluating HOPE-modalities versus SCS, the only use of dHOPE displayed a significant risk reduction (HR=0.391, 95% CI: 0.214–0.714, *P*=0.002; Fig. 2F), while this was not observed for use of sHOPE (HR=0.687, 95% CI: 0.387–1.222, *P*=0.202; Fig. 2F). In regards to overall survival or death-censored graft survival, no difference was observed when comparing SCS to HOPE (Supplementary Figure 3A/C, Supplemental Digital Content 2, http://links.lww.com/JS9/D515) or HOPE-modalities (Supplementary Figure 3B/D, Supplemental Digital Content 2, http://links.lww.com/JS9/D515). Further, no difference in the incidence of retransplantations after the first successful OLT was observed after HOPE (Supplementary Figure 3E, Supplemental Digital Content 2, http://links.lww.com/JS9/D515) or in regards to HOPE-modalities (Supplementary Figure 3F, Supplemental Digital Content 2, http://links.lww.com/JS9/D515) when compared to SCS.

Ultimately, a competing risk analysis for time to first biliary complication, mortality and retransplantation was conducted. However, no effect of preservation strategies (either SCS vs HOPE or SCS vs dHOPE vs sHOPE) on cumulative incidences of mortality or retransplantation were observed, and both potentially competing events were not associated with the observations made in regards to biliary complications during follow-up (see Supplementary Figure 4, Supplemental Digital Content 2, http://links.lww.com/JS9/D515).

## Discussion

We here present the first analysis of real world data comparing modalities of HOPE with SCS in regards to outcome after OLT. While perfusion via portal vein alone or additional use of arterial perfusion for HOPE is commonly considered a decision made by each center, we observed a specifically beneficial effect of dHOPE on patient outcome and the occurrence of biliary complications in particular. Biliary complications are long known to be the Achilles heel of OLT, which is underlined by their high occurrence in approximately one-fifth of benchmark cases^4^. Pathophysiologically the biliary tree and its epithelial cells are most sensitive to oxygen deprivation and ischemia-reperfusion injury (IRI)^13^. Biliary complications include both short-term events like biliary leakage and long-term manifestations, that is, ischemic-type cholangiopathies. Thereby, the probability of biliary complications affects patients at any stage after OLT and their avoidance is central in order to improve patients’ outcome.

As evaluated in randomized controlled trials^2^, our study showed that patients undergoing OLT with HOPE-treated organs display a reduced incidence of biliary complications. Importantly, most patients in the present cohort received liver grafts which were donated after brain death and could be referred to as standard criteria organs. Still, an effect on biliary complications, which was previously mainly explored in marginal liver grafts could be documented. In particular, HOPE reduced the need for surgical revision in case of biliary complications when compared to SCS (24.6% vs 11.8%). Of note, while the decision for surgical revision for biliary complications is not standardized in the field of OLT, a reduction of overall biliary complications after the use of HOPE was observed in this cohort. This further translated into shorter overall hospital stays in the HOPE cohort. Moreover, a prolongation of time until the first biliary complication could be appreciated in HOPE cases. Both the association of dHOPE with reduced risk for severe biliary complications, as well as with time to first biliary complications, remained independent from confounders as evaluated via appropriate models. While the absolute reduction of these complications is vital, a shift towards a later and potentially more stable time point after OLT seems to be desirable. Noticeably, overall survival did not significantly differ between cohorts, while a tendency towards improved survival was observed for patients receiving HOPE-treated liver grafts. The rate of retransplantations and death-censored graft survival were comparable between cohorts. This is paralleled by a comparable incidence of EAD between SCS and HOPE cohorts. Accordingly, while the presented data underlines a protective effect of HOPE in regards to biliary injury, an improvement in outcome parameters of liver function and long-term graft viability cannot be observed. Commonly, HOPE is known to replenish intracellular ATP stores and thereby reduce the production of reactive oxygen species after reperfusion^6^. Extracellular ATP acts as a proinflammatory messenger and driver of IRI while adenosine displays protective immunomodulatory properties^19^. Reduction of extracellular ATP release and conversion of extracellular ATP to adenosine might be promoted during HOPE and ultimately contribute to its known effect in the reduction of inflammation and IRI^20^. The reported findings in regards to EAD development and postoperative parameters of liver function might be explained by the robustness of hepatocytes due to their polyploidy on the one hand and by the vast potential for regeneration harbored by the hepatocellular compartment, which in case of an acute injury mainly relies on hepatocellular replication rather than activation of the hepatic precursor cell niche as a subset of cholangiocytes^21,22^. Cholangiocytes, however, are highly sensitive to oxidative stress and easily affected by perturbation in hemodynamics and oxygen supply while also being exposed to a potentially toxic environment due to their location and objectives in the hepatic microanatomy^23^. Accordingly, preservation of liver grafts via SCS might be sufficient for safeguarding of hepatocellular function, while additional effort needs to be made in order to preserve cholangiocytes and ensure their regeneration after IRI^13^. Yet, when exploring the duration of perfusion (≤/> 150 min) in subgroups of sHOPE or dHOPE-treated liver grafts, a potentially protective effect for EAD development was observed for longer perfusion periods when using dHOPE (Supplementary Figure 5, Supplemental Digital Content 2, http://links.lww.com/JS9/D515). Indeed, a difference in incidence of EAD of 45.5% versus 15.2% could be observed, leading to the suggestion that not only the cholangiocellular, but also the hepatocellular compartment might profit from dHOPE when applying longer periods of perfusion.

Most importantly, the present study was able to gather further insight in differential effects of sHOPE and dHOPE in a real-world cohort of patients undergoing OLT at the same institution. There is no prospective data on the direct comparison of both HOPE-modalities available until today. Regardless of the lack of supporting data, it was previously suggested that this matter has only little implication for patient outcome^13^. In our cohort, both HOPE-modalities seem to exert similar effects on development and severity of biliary complications, while not affecting incidence of EAD and graft survival. However, only use of dHOPE ultimately proved to be independently associated with reduction of surgical revisions for biliary complications upon multivariable logistic regression analysis. Pathomechanistically, HOPE was associated with improved biliary viability due to reduced vascular damage and subsequently improved oxygenation and perfusion^24,25^. Yet, experimental and translational data on the direct comparison of HOPE-modalities is vastly lacking to date. In this context, one preclinical model reported by de Vries *et al*.^26^ showed a significant reduction of hepatocellular and cholangiocellular injury as depicted by lower circulating ALT and bile LDH 4 h after OLT when using dHOPE versus sHOPE in pigs. In addition, a lower expression of the vasoconstrictor endothelin-1 in the hepatic artery of dHOPE-treated organs could be documented. Accordingly, dHOPE might promote optimized oxygen supply to the biliary epithelium and thereby lead to improved protection from IRI. In fact, a contribution of disturbed arterial perfusion and oxygenation to the development of biliary complications was discussed already early on in transplantation research^27^. While an intact perfusion via the portal system might allow for the preservation of the hepatic lobule, arterial perfusion seems to be crucial for the viability of the biliary tract. In addition, hepatic sinusoidal endothelial cells were recently described as key players during IRI and machine perfusion^28,29^. Hence, additional perfusion via the hepatic artery might directly affect sinusoidal hemodynamics and thereby induce the observed beneficial effects. Moreover, a recently published translational study by Rauter *et al*.^30^ revealed a potential association of endothelial glycocalyx damage on development of adverse outcome after OLT. In fact, prolonged perfusion using HOPE was associated with an increase in glycocalyx degradation as evaluated via syndecan-1 concentrations in the perfusate. However, when directly comparing sHOPE and dHOPE, the latter seemed to preserve organs from increased damage during prolonged preservation, again underlining a potentially protective effect of arterial perfusion and oxygenation on the endothelial compartment.

The presented data encourages the application of dHOPE whenever possible, mainly due to an improved preservation of the biliary compartment and associated reduction in severe biliary complications. Yet, there are certain limitations that need to be mentioned. Firstly, the evaluated follow-up period of a median of 19 months might not allow for investigation of all biliary complications occurring in this cohort, as ischemic-type cholangiopathies can be observed even after this interval. Nevertheless, the reported follow-up is longer when compared to available data on prospective trials so far^2^. One of the most relevant limitations of this study is the selection of HOPE-modality based on individual decisions. Of note, dHOPE is applied as a standard method for hypothermic perfusion at our center, while sHOPE is used as a viable option for grafts with unsuitable vessels for arterial canulation. This included a lack of an aortic patch (e.g. simultaneous donation of the pancreas) or multiple arteries and the need for back table reconstruction. In association with the second limitation posed by the retrospective single-center design of this study, evaluation of an underlying selection bias is virtually impossible. However, while donor demographics seem to be evenly distributed across HOPE-modalities, the impact of arterial vessel suitability on patient outcome can ultimately not be evaluated. Accordingly, the observations made in this study need to be addressed and validated in large randomized controlled trials in order to allow for robust decision making on whether or whether not to use dHOPE routinely.

In conclusion, this analysis of a large series of patients undergoing OLT in a real world setting underlines a beneficial effect of HOPE. HOPE leads to reduced biliary complications, surgical revisions and shorter hospitalization, thereby impacting both patient management and hospital costs. When compared to other organ perfusion methods under current investigation, HOPE is a simple method for preconditioning of liver grafts, as it can be performed in parallel to recipient hepatectomy.

## Ethical approval

This study was approved by the ethics committee of Medical University of Vienna (EK# 1610/2023).

## Consent

While we only report on a retrostpective data analysis, informed consent was obtained from all included individuals.

## Source of funding

No funding was received for this work.

## Author contribution

D.P.: conceptualization, data curation, formal analysis, investigation, methodology, project administration, supervision, validation, visualization, writing of original draft, and review and editing; J.D.: conceptualization, data curation, formal analysis, investigation, methodology, supervision, validation, visualization, and review and editing; M.R. and S.K.: data curation, project administration, and review and editing; L.R., N.B., T.S.Z., and C.T.: data curation, validation, and review and editing; P.S.: validation, writing of original draft, and review and editing; R.R.: investigation, methodology, software, visualization, and review and editing; G.S., A.S., and T.S.: validation, review, and editing; G.B. and G.G.: conceptualization, investigation, resources, software, supervision, validation, writing of original draft, and review and editing.

## Conflicts of interest disclosure

The authors declare no conflicts of interest.

## Research registration unique identifying number (UIN)

1. Name of the registry: ClinicalTrials.gov.
2. Unique identifying number or registration ID: NCT06418165.
3. Hyperlink to your specific registration (must be publicly accessible and will be checked): https://www.clinicaltrials.gov/study/NCT06418165?cond=Liver&term=VIHOMPS&rank=1.

## Guarantor

David Pereyra, Georg Györi, and Gabriela Berlakovich.

## Data availability statement

All data is available upon reasonable request to the corresponding author.

## Provenance and peer review

Not commissioned, externally peer-reviewed.

## Supplementary Material

SUPPLEMENTARY MATERIAL
