# Supplementary material for: Dual hypothermic oxygenated machine perfusion of the liver reduces post-transplant biliary complications: a retrospective cohort study
Source: Int J Surg. 2024 Oct 18;110(12):7909–18. doi: 10.1097/JS9.0000000000002115 (PMC11634107; doi:10.1097/JS9.0000000000002115)
Supplement: SUPPLEMENTARY MATERIAL [file js9-110-7909-s002.docx]

Supplementary Figures

*Supplementary Figure 1. Visualization of differences in baseline demographics.*

1. Body-mass-index is shown according to type of preservation. Further, **B)** prevalence of cardiovascular diseases and **C)** use of liver grafts from donation after cardiac death is visualized. Similarly, **D)** donor gamma-glutamyl transferase is shown according to type of preservation. * p<0.05

*Supplementary Figure 2. Comparison of Biliary Complications without Biliary Leaks*

**A)** Incidence of all biliary complications (p = 0.095), biliary complications with need for surgical revision (p = 0.261) and biliary complications which were treated via interventional endoscopic retrograde cholangio-pancreatography (ERCP, p = 0.201) are compared between organs subjected to static cold storage (SCS) or hypothermic oxygenated machine perfusion (HOPE). **B)** Similarly, comparison between SCS and HOPE-modalities (single HOPE = sHOPE, dual HOPE = dHOPE) was performed. Incidence of all biliary complications (SCS vs HOPE: p = 0.461, SCS vs dHOPE: p = 0.039, sHOPE vs dHOPE: p = 0.190), biliary complications with need for surgical revision (SCS vs HOPE: p = 0.930, SCS vs dHOPE: p = 0.066, sHOPE vs dHOPE: p = 0.066) and biliary complications which were treated via interventional ERCP (SCS vs HOPE: p = 0.533, SCS vs dHOPE: p = 0.396, sHOPE vs dHOPE: p = 0.133) is visualized.

*Supplementary Figure 3. HOPE does not affect overall survival and graft survival.*

Overall survival is shown for patients after **A)** SCS vs HOPE (mean in months: SCS = 43.2, 95%CI = 36.4-50.0; HOPE = 49.5, 95%CI = 45.9-53.1, log-rank: p=0.050) and according to **B)** HOPE-modalities (mean in months: sHOPE = 40.7, 95%CI = 36.3-45.0; dHOPE = 50.0, 95%CI = 45.3-54.7, log-rank: p=0.142). Death-censored graft survival is shown for patients after **C)** SCS vs HOPE (mean in months: SCS = 51.9, 95%CI = 46.1-57.6; HOPE = 54.1, 95%CI = 51.2-57.0, log-rank: p=0.305) and according to **D)** HOPE-modalities (mean in months: sHOPE = 44.4, 95%CI = 40.8-48.0; dHOPE = 54.6, 95%CI = 50.8-58.3, log-rank: p=0.585). Further, incidence of re-transplantation is shown for **E)** SCS versus HOPE (3 of 69 [4.3%] in SCS vs 9 of 173 [5.2%] in HOPE, p=0.782) as well as **F)** or in regards to HOPE-modalities (5 of 73 [6.8%] in sHOPE vs 4 of 100 [4.0%] in dHOPE; SCS vs sHOPE: p=0.518, SCS vs dHOPE: p=0.911, sHOPE vs dHOPE: p=0.405).

B

A

*Supplementary Figure 4. Competing risk analysis for biliary complication, re-transplantation and death.*

A) Cumulative incidence curves for biliary complication, re-transplantation and death after SCS and HOPE. Cumulative incidence of the considered competing events is shown for patients after static cold storage (SCS) and hypothermic oxygenated machine perfusion (HOPE). Cumulative incidence regarding biliary complication was significantly larger under SCS compared to HOPE, p=0.012. Differences between SCS and HOPE regarding retransplantation or death were not significant (p=0.165 and p=0.298, respectively). B) Cumulative incidence curves for biliary complication, re-transplantation and death after SCS, sHOPE and dHOPE. Cumulative incidence of the considered competing events is shown for patients after static cold storage (SCS) and hypothermic oxygenated machine perfusion (sHOPE and dHOPE). Cumulative incidence regarding biliary complication was significantly different between the three groups, p=0.012. Differences betweenthe groups regarding retransplantation or death were not significant (p=0.377 and p=0.583, respectively).

*Supplementary Figure 5. Impact of perfusion time on outcome.*

A) Incidence of early allograft dysfunction (EAD), biliary complications, and biliary complications with relevance of surgical revision were evaluated according to perfusion time of ≤/> 150 minutes. Subgroup analysis was performed for B) single HOPE (sHOPE) and for C) dual HOPE (dHOPE). P-values are reported in the figure.

Supplementary Tables

| **Supplementary Table 1. Perioperative Laboratory Parameters (Median, Interquartile Range)** | | | | | | |
| --- | --- | --- | --- | --- | --- | --- |
| **Parameter** | **SCS**  **(N = 69)** | **sHOPE**  **(N = 76)** | **dHOPE**  **(N = 102)** | ***p-value^1^*** | ***p-value^2^*** | ***p-value^3^*** |
| Haemoglobin (mg/dL)  Pre-OP | 10.4  (9.0-12.0) | 11.3  (8.9-12.5) | 10.9  (8.9-12.9) | 0.394 | 0.415 | 0.997 |
| Haemoglobin (mg/dL)  POD1 | 8.5  (8.0-9.9) | 9.4  (8.7-10.7) | 9.3  (8.4-10.0) | 0.029 | 0.081 | 0.274 |
| Haemoglobin (mg/dL)  POD7 | 8.5  (7.8-9.4) | 8.5  (7.9-9.5) | 8.4  (7.7-9.4) | 0.624 | 0.672 | 0.351 |
| Haemoglobin (mg/dL)  POD14 | 8.3  (7.8-9.3) | 8.5  (7.6-9.4) | 8.3  (7.7-9.0) | 0.740 | 0.373 | 0.233 |
| Platelet count (G/L)  Pre-OP | 92  (68-141) | 94  (73-129) | 97  (60-145) | 0.958 | 0.593 | 0.773 |
| Platelet count (G/L)  POD1 | 58  (43-91) | 64  (39-91) | 66  (45-104) | 0.975 | 0.380 | 0.468 |
| Platelet count (G/L)  POD7 | 70  (52-104) | 84  (51-118) | 79  (59-119) | 0.365 | 0.377 | 0.893 |
| Platelet count (G/L)  POD14 | 212  (129-322) | 212  (128-291) | 245  (152-292) | 0.635 | 0.930 | 0.306 |
| Creatinine (mg/dL)  Pre-OP | 0.94  (0.73-1.22) | 0.92  (0.81-1.11) | 0.93  (0.80-1.27) | 0.866 | 0.556 | 0.385 |
| Creatinine (mg/dL)  POD1 | 1.15  (0.91-1.39) | 1.22  (0.99-1.73) | 1.28  (0.98-1.68) | 0.305 | 0.115 | 0.785 |
| Creatinine (mg/dL)  POD7 | 1.21  (0.79-1.80) | 1.07  (0.82-1.68) | 1.18  (0.83-2.07) | 0.550 | 0.899 | 0.418 |
| Creatinine (mg/dL)  POD14 | 1.18  (0.76-1.55) | 1.13  (0.80-1.66) | 1.17  (0.84-1.67) | 0.896 | 0.555 | 0.453 |
| Bilirubin (mg/dL)  Pre-OP | 1.78  (1.02-4.06) | 2.33  (1.22-4.49) | 2.21  (1.05-4.27) | 0.288 | 0.838 | 0.406 |
| Bilirubin (mg/dL)  POD1 | 4.08  (2.58-7.53) | 4.33  (2.47-6.80) | 4.55  (2.45-6.99) | 0.912 | 0.955 | 0.943 |
| Bilirubin (mg/dL)  POD7 | 2.78  (1.15-7.84) | 3.18  (1.78-8.78) | 2.53  (1.41-6.11) | 0.395 | 0.992 | 0.243 |
| Bilirubin (mg/dL)  POD14 | 1.52  (0.82-4.59) | 1.87  (0.98-3.06) | 1.32  (0.74-2.71) | 0.584 | 0.324 | 0.060 |
| ALP (U/L)  Pre-OP | 125  (88-197) | 121  (85-186) | 150  (88-221) | 0.891 | 0.540 | 0.598 |
| ALP (U/L)  POD1 | 70  (59-90) | 80  (59-115) | 82  (56-125) | 0.310 | 0.311 | 0.984 |
| ALP (U/L)  POD7 | 132  (87-216) | 161  (122-213) | 156  (114-188) | 0.172 | 0.633 | 0.288 |
| ALP (U/L)  POD14 | 148  (85-244) | 133  (94-204) | 125  (91-186) | 0.643 | 0.384 | 0.538 |
| GGT (U/L)  Pre-OP | 122  (53-219) | 89  (54-163) | 97  (60-185) | 0.302 | 0.802 | 0.420 |
| GGT (U/L)  POD1 | 72  (36-112) | 67  (43-117) | 84  (52-145) | 0.863 | 0.237 | 0.210 |
| GGT (U/L)  POD7 | 328  (172-463) | 351  (235-512) | 363  (202-572) | 0.140 | 0.272 | 0.799 |
| GGT (U/L)  POD14 | 208  (117-315) | 225  (123-332) | 187  (131-286) | 0.446 | 0.974 | 0.477 |
| AST (U/L)  Pre-OP | 52  (36-85) | 65  (38-154) | 56  (39-117) | 0.251 | 0.297 | 0.720 |
| AST (U/L)  POD1 | 953  (546-1725) | 773  (454-2015) | 817  (415-1590) | 0.536 | 0.513 | 0.913 |
| AST (U/L)  POD7 | 63  (41-131) | 76  (48-103) | 65  (46-94) | 0.417 | 0.907 | 0.248 |
| AST (U/L)  POD14 | 31  (20-43) | 30  (19-42) | 26  (17-41) | 0.925 | 0.418 | 0.324 |
| ALT (U/L)  Pre-OP | 40  (22-65) | 43  (26-117) | 43  (26-69) | 0.137 | 0.359 | 0.529 |
| ALT (U/L)  POD1 | 561  (416-1159) | 500  (318-1094) | 486  (239-1153) | 0.412 | 0.350 | 0.595 |
| ALT (U/L)  POD7 | 204  (127-394) | 170  (123-309) | 168  (100-280) | 0.515 | 0.078 | 0.219 |
| ALT (U/L)  POD14 | 69  (46-109) | 63  (46-107) | 64  (35-95) | 0.646 | 0.182 | 0.328 |
| INR  Pre-OP | 1.5  (1.3-1.8) | 1.5  (1.3-1.8) | 1.5  (1.3-1.7) | 0.942 | 0.675 | 0.578 |
| INR  POD1 | 1.8  (1.5-2.1) | 1.7  (1.5-2.0) | 1.8  (1.5-2.1) | 0.603 | 0.868 | 0.525 |
| INR  POD7 | 1.3  (1.2-1.4) | 1.2  (1.1-1.4) | 1.3  (1.2-1.4) | 0.400 | 0.952 | 0.314 |
| INR  POD14 | 1.1  (1.1-1.2) | 1.1  (1.1-1.3) | 1.1  (1.1-1.3) | 0.759 | 0.714 | 0.976 |
| SCS = static cold storage, HOPE = hypothermic oxygenated machine perfusion, ALP = alkaline phosphatase, GGT = gama-glutamyltransferase, AST = aspartate aminotransferase, ALT = alanine aminotransferase, INR = international normalized ratio; p-values: ^1^ SCS vs sHOPE, ^2^ SCS vs dHOPE, ^3^ sHOPE vs dHOPE. | | | | | | |

| **Supplementary Table 2.**  **Extended Multivariable Analysis with Stepwise Forward Exclusion**  **for Biliary Complication with Surgical Intervention** | | | | | | |
| --- | --- | --- | --- | --- | --- | --- |
|  | Univariate Analysis | | | Multivariable Analysis | | |
| Parameter | OR | 95%CI | p-value | OR | 95%CI | p-value |
| Use of dHOPE | 0.326 | 0.143 – 0.745 | 0.008 | **0.326** | **0.143 – 0.745** | **0.008** |
| Recipient characteristics |  |  |  |  |  |  |
| BMI (kg/m^2^) | 1.000 | 0.931 – 1.074 | 0.995 | 1.007 | 0.931 – 1.090 | 0.861 |
| Cardiovascular diseases | 1.184 | 0.580 – 2.419 | 0.643 | 0.972 | 0.441 – 2.143 | 0.944 |
| Donor characteristics |  |  |  |  |  |  |
| DCD | 3.804 | 1.020 – 14.191 | 0.047 | 2.871 | 0.752 – 10.957 | 0.123 |
| GGT (U/L) | 1.000 | 0.997 – 1.004 | 0.912 | 1.001 | 0.998 – 1.005 | 0.556 |
| Cold Ischemia (min) | 1.002 | 0.999 – 1.005 | 0.129 | 1.001 | 0.998 – 1.004 | 0.609 |
| OR = Odds ratio, 95%CI = 95% confidence interval, HOPE = hypothermic oxygenated machine perfusion, BMI = body mass index, DCD = donation after circulatory death, GGT = gama-glutamyltransferase | | | | | | |

| **Supplementary Table 3. Multivariable Analysis for Biliary Complications** | | | | | | |
| --- | --- | --- | --- | --- | --- | --- |
|  | Univariate Analysis | | | Multivariable Analysis | | |
| Parameter | OR | 95%CI | p-value | OR | 95%CI | p-value |
| Use of sHOPE | 1.069 | 0.583 – 1.962 | 0.829 |  |  |  |
| **Use of dHOPE** | **0.477** | **0.260 – 0.877** | **0.017** | **0.481** | **0.257 – 0.902** | **0.022** |
| Perfusion Time (min)* | 1.000 | 0.996 – 1.004 | 0.873 |  |  |  |
| **Recipient characteristics** |  |  |  |  |  |  |
| Male | 1.204 | 0.618 – 2.346 | 0.585 |  |  |  |
| Age (years) | 0.993 | 0.968 – 1.017 | 0.553 |  |  |  |
| MELD (points) | 1.036 | 0.995 – 1.078 | 0.085 |  |  |  |
| MELD-Na (points) | 1.036 | 0.997 – 1.077 | 0.073 |  |  |  |
| BMI (kg/m^2^) | 1.002 | 0.945 – 1.063 | 0.948 |  |  |  |
| First OLT | 0.671 | 0.183 – 2.455 | 0.546 |  |  |  |
| **HU-OLT** | **3.429** | **1.191 – 9.874** | **0.022** | **3.505** | **1.170 – 10.497** | **0.025** |
| Cardiovascular diseases | 0.987 | 0.549 – 1.776 | 0.966 |  |  |  |
| Arterial hypertension | 0.686 | 0.368 – 1.276 | 0.234 |  |  |  |
| Diabetes type II | 1.303 | 0.643 – 2.638 | 0.463 |  |  |  |
| Smoking | 0.635 | 0.248 – 1.625 | 0.343 |  |  |  |
| Ascites | 0.590 | 0.315 – 1.107 | 0.100 |  |  |  |
| Encephalopathy | 1.093 | 0.510 – 2.343 | 0.819 |  |  |  |
| Hepatorenal syndrome | 0.459 | 0.130 – 1.622 | 0.227 |  |  |  |
| **Chronic kidney disease** | **0.230** | **0.068 – 0.781** | **0.018** | **0.271** | **0.079 – 0.932** | **0.038** |
| Pulmonary disease | 1.355 | 0.667 – 2.752 | 0.400 |  |  |  |
| **Donor characteristics** |  |  |  |  |  |  |
| Male sex | 1.113 | 0.629 – 1.970 | 0.713 |  |  |  |
| DCD | 2.738 | 0.766 – 9.786 | 0.121 |  |  |  |
| Age (years) | 1.001 | 0.983 – 1.019 | 0.936 |  |  |  |
| BMI (kg/m^2^) | 0.948 | 0.881 – 1.019 | 0.146 |  |  |  |
| Smoking | 1.295 | 0.547 – 3.067 | 0.557 |  |  |  |
| Diabetes type II | 0.715 | 0.209 – 2.442 | 0.592 |  |  |  |
| Alcohol abuse | 0.523 | 0.179 – 1.526 | 0.236 |  |  |  |
| DRI | 1.000 | 0.998 – 1.001 | 0.725 |  |  |  |
| ET-DRI | 0.951 | 0.302 – 2.993 | 0.931 |  |  |  |
| GGT (U/L) | 0.998 | 0.995 – 1.002 | 0.330 |  |  |  |
| Steatosis (%) | 0.977 | 0.948 – 1.007 | 0.137 |  |  |  |
| Cold Ischemia (min) | 1.002 | 0.999 – 1.004 | 0.165 |  |  |  |
| OR = odds ratio, 95%CI = 95% confidence interval, HOPE = hypothermic oxygenated machine perfusion, MELD = model of end-stage liver disease, Na = sodium, BMI = body mass index, OLT = orthotopic liver transplantation, HU = high urgency, DCD = donation after cardiac death, DRI = donor risk index, ET = Eurotransplant, GGT = gama-glutamyltransferase.  * Univariate analysis of perfusion time is restricted to HOPE cohort (i.e., sHOPE and dHOPE) | | | | | | |
